# Possible Concomitant Aggressive NK Cell Leukemia and EBV-positive T-cell lymphoma; Using the online beta version of WHO-HAEM5 and videoconferencing software to make diagnoses accessible in an emerging economy

**DOI:** 10.1186/s13000-023-01395-9

**Published:** 2023-10-06

**Authors:** Vanessa J. Dayton, Dang Hoang Thien, Phan Thi Xinh, Cade Arries, Nguyen Ngoc Sang, Ngo Ngoc Ngan Linh, Nguyen Phuong Lien, Phu Chi Dung

**Affiliations:** 1 Laboratory Medicine and Pathology, Hennepin County Medical Center, Minneapolis, MN USA; 2 Laboratory Medicine and Pathology, University of Minnesota Medical Center-Fairview and University of Minnesota Medical School, Hematopathology, Minneapolis, MN USA; 3 Blood Transfusion Hematology Hospital, Ho Chi Minh City, Vietnam

**Keywords:** WHO-HAEM5 classification (beta version online), Aggressive NK Cell leukemia, chronic active EBV disease, Systemic EBV-positive T-cell lymphoma of Childhood, Hemophagocytic lymphohistiocytosis, *UNC13D*, Telepathology, Diagnostic hematopathology in emerging economies

## Abstract

**Background:**

Using the World Health Organization Classification 5th edition (beta version online; WHO-HAEM5bv) in emerging economies is key to global healthcare equity. Although there may be ongoing updates, hesitancy in accepting and reporting these diagnoses in publication conflicts with the WHO’s commitment to global accessibility. Aggressive NK cell leukemia (ANKL) and systemic EBV-positive T-cell lymphoma of childhood (SEBVTCL) with CD4-positive immunophenotype are both rare entities, are most described in Asians and East Asians, are associated with prior systemic chronic active EBV disease (CAEBV), and presentation with Hemophagocytic Lymphohistiocytosis (HLH). Recognizing and diagnosing any one of these entities requires not only training and experience in hematopathology, but good cooperation between clinical physicians and all areas of the laboratory. We describe a 30-year-old woman who presented to a Vietnam hospital and was rapidly diagnosed with ANKL, SEBVTCL, and HLH using WHO-HAEM5bv essential criteria, aided by expert consultation from a United States (US) board certified hematopathologist in real-time using video conferencing software.

**Methods:**

Zoom™ videoconferencing software; Immunohistochemistry; flow cytometric immunophenotyping; polymerase chain reaction (PCR), Next Generation Sequencing (NGS).

**Results:**

At the time of hospital admission, automated complete blood count (CBC) with differential count showed slight anemia, slight lymphocytosis, and moderate thrombocytopenia. HIV serology was negative. Whole blood PCR for EBV was positive showing 98,000 copies/ml. A lymph node biopsy revealed histology and immunohistochemistry consistent with the online beta version WHO-HAEM5 classification of SEBVTCL arising in CAEBV. Blood and bone marrow studies performed for staging revealed no histologic or immunohistochemical evidence of T-cell lymphoma in the bone marrow core, however, atypical blood smear lymphocyte morphology and blood immunophenotyping by flow cytometry were consistent with WHO-HAEM5 classification of ANKL. NGS revealed no evidence of genetic variant(s) associated with HLH in Vietnam. All laboratory studies were performed at Blood Transfusion Hematology Hospital (BTHH) in Ho Chi Minh City Vietnam.

**Conclusion:**

Although Vietnam, an emerging economy, currently lacks the laboratory infrastructure to more rigorously confirm a rare synchronous presentation of two distinct EBV-driven T/NK cell neoplasms, these two concomitant diagnoses were made using only laboratory techniques available in Vietnam with the help of WHO-HAEM5bv and real-time video consultation by a US hematopathologist.

## Introduction

A beta-version of the 5th edition of the World Health Organization (WHO) classification of hematopoietic neoplasms was made available in a web-based format in late 2022 (WHO-HAEM5bv) [1]. This edition describes both aggressive NK cell leukemia (ANKL) and systemic EBV-positive T-cell lymphoma of childhood (SEBVTCL) as being rare, as being more common in Asians, and as commonly being associated with hemophagocytic lymphohistiocytosis (HLH) [2, 3]. Both are described as, in some cases, evolving from systemic chronic active EBV disease (CAEBV). Whereas ANKL is grouped with mature T-cell and NK-cell leukemias and lymphomas, and is described as being a disease seen in adults, both CAEBV and SEBVTCL are grouped under the heading of EBV-positive T-cell and NK-cell lymphoid proliferations and lymphomas of childhood [4, 5]. The goal of this brief report is to review the clinical, laboratory, and morphologic findings that are helpful in suspecting, recognizing and diagnosing these entities while emphasizing the role that real-time consultation with experts in developed countries using video conferencing software can play, along with WHO-HAEM5bv, to making these difficult diagnoses accessible in a resource limited setting.

## Case report

### Clinical history

A 30-year-old Vietnamese female with no history of immunodeficiency had been followed closely by her primary care physician for one year due to persistent infectious mononucleosis (IM) symptoms following an episode of serology confirmed acute EBV pharyngitis. Over the course of two weeks, she noted acute onset of high fever, painful cervical and axillary lymphadenopathy, abdominal fullness, and generalized petechiae. She was admitted to a hospital in Ho Chi Minh City, Vietnam, that specializes in hematologic disease, where a standard workup was diagnostic for HLH (Table 1), and with whole blood PCR for EBV positive for 98,000 copies/ml. HIV, hepatitis B, and hepatitis C serologies were negative. CT scan of the abdomen revealed marked hepatosplenomegaly and abdominal lymphadenopathy. Next Generation Sequencing (NGS) performed for genetic variants associated with congenital HLH in the Vietnamese population, including *UNC13D*, was negative [6]. A cervical lymph node biopsy, unilateral bone marrow biopsy with attempted marrow aspiration and blood smear review, and flow cytometric immunophenotyping of blood were performed.

**Table 1:** Patient clinical and laboratory parameters at time of hospital admission

Clinical and Laboratory Parameters	Patient at time of Hospital admission	Laboratory Normal Range	WHO-HAEM5bv HLH Diagnostic Criteria*
Body Temperature	39.0 C		> 38.5 C
Spleen Size	18 cm		> 3 cm below costal margin
Automated Complete Blood Count (CBC) Hemoglobin (Hgb) Absolute neutrophils (Abs neut’s) Platelets (Plt)	11.2 g/dL 2.26 k/µL 67 k/µL	12.0-15.6 g/dL 1.5–7.7 k/µL 150–370 l/µL	Cytopenias in > 2 cell lines as below** Hgb < 9gm/dL Abs Neut’s < 0.1k/µL Plt < 100,000/µL
Hemophagocytosis identified on tissue Smears or sections	Identified in lymph node sections		Present
NK Cell Activity	1.3%	81–100%***	Low
Serum Ferritin	1369.24 ng/ml	4.6–204 ng/ml	> 500 ng/ml
CD25 level (soluble interleukin)	NA	NA	> 2.0 mMol/L
Fasting serum Triglyceride Fibrinogen	2.43 mMol/L 134 g/dL	< 1.7 mMol/L 200–400 g/dL	Triglyceride > 2.0 mMol/L *or* Fibrinogen < 150 mg/dL

*WHO-HAEM5bv requires 5 of the 8 criteria satisfied for a diagnosis of Hemophagocytic Lymphohistiocytosis (HLH) [3]

** Cytopenias in > 2 cells lines count as fulfilling one criterion

*** CD107a measured by flow cytometry method

NA = Not available

### Pathologic findings

**Fig. 1:**
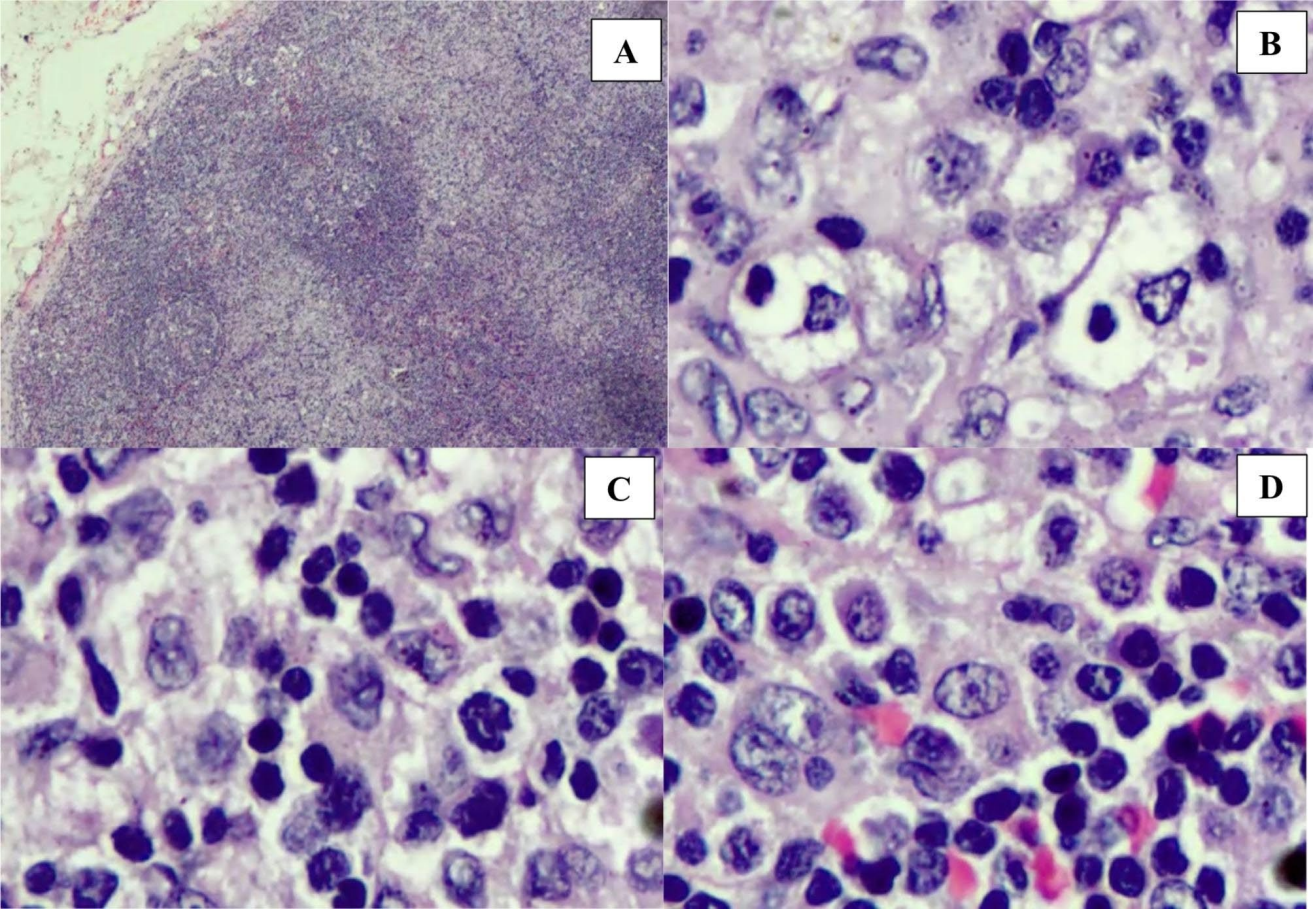
Images of H&E stain, lymph node sections; (**A**) Low power image showing attenuated follicles with depleted germinal centers and marked expansion of the paracortex; (**B**) High power image of paracortical infiltrate including hemophagocytic histiocytes; (**C**) High power image of paracortex showing background small lymphocytes and more predominant large and atypical lymphoid cells; (**D**) High power image of paracortex showing large atypical lymphoid cells including a binucleate cell

**Fig. 2:**
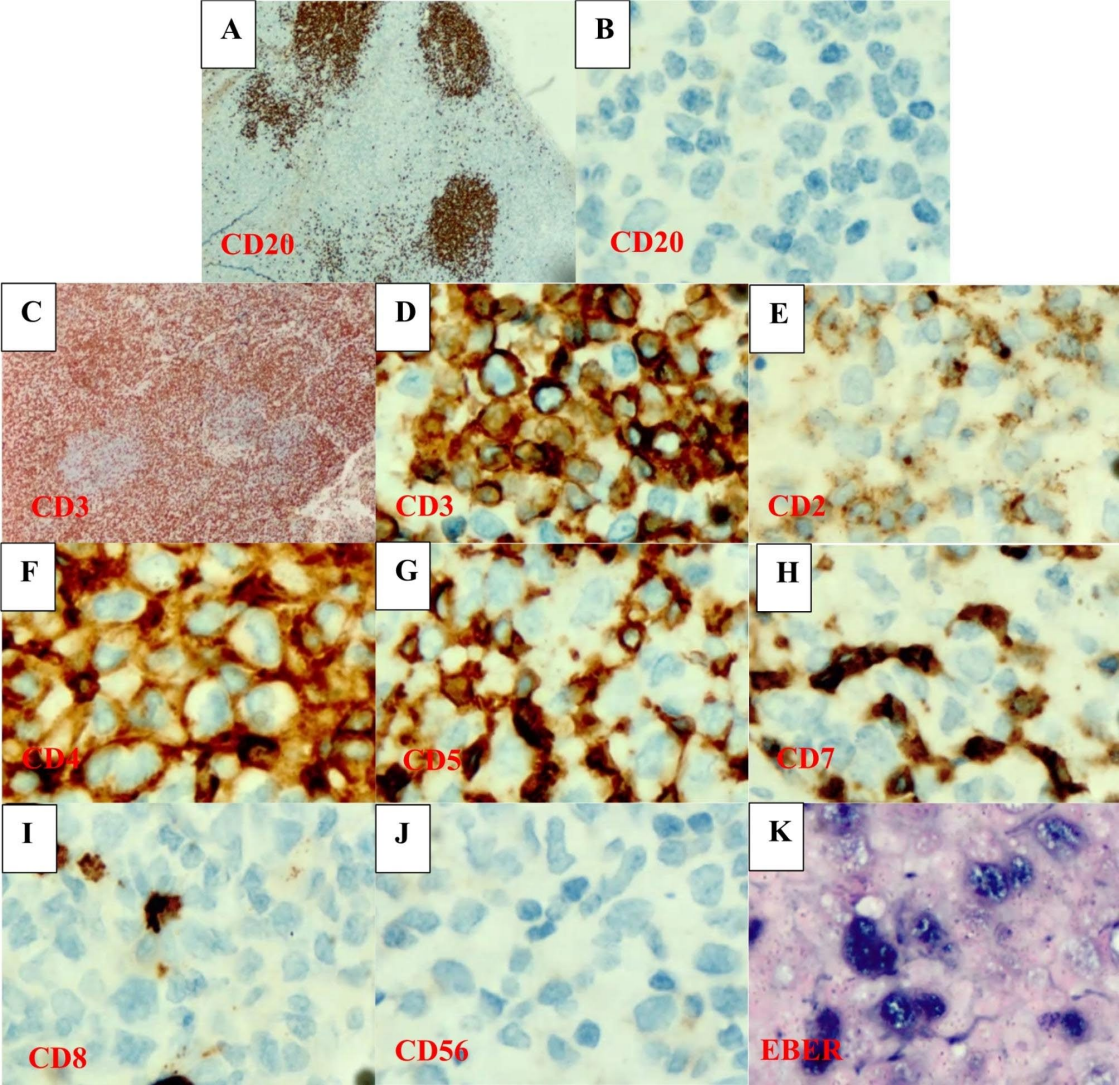
Immunostains and EBER-ish for lymph node sections; **A**. CD20 low power highlights follicles; **B**. CD20 high power in paracortex highlights rare small B-cells; **C**. CD3 low power highlights paracortical expansion by T-cells; **D**. CD3 high power in paracortex highlights small T-cells and large atypical cells; **E**. CD2 high power in paracortex shows dark staining of small lymphs and weak/partial staining of large cells; **F**. CD4 in paracortex highlights small and large cells; **G**. CD5 in paracortex shows weak, variable expression in large cells; **H**. CD7 in paracortex highlights few small lymphocytes; **I**. CD8 in paracortex highlights few small lymphocytes; **J**. CD56 in paracortex is negative in small and large lymphoid cells; **K**. EBER-ish in paracortex highlights large cells and few small lymphocytes

The lymph node biopsy showed few, attenuated follicles with depleted germinal centers and marked paracortical expansion by a mixed population of histiocytes, including hemophagocytic histiocytes, small lymphocytes and larger, atypical appearing lymphoid cells (Fig. 1). Immunohistochemical staining for CD20 confirmed the presence of attenuated follicles and revealed rare to absent small B-lymphocytes in areas of paracortical expansion. CD3 staining revealed a predominant T-cell population in the expanded paracortex with dark surface staining of the small lymphocytes and weaker surface staining of the larger and atypical lymphoid cells (Fig. 2, A-D). Additional stains revealed that the larger lymphoid cells in areas of paracortical expansion showed weak staining for CD2, uniform staining for CD4, variable staining for CD5 and CD7, and no staining for CD8 or CD56 (Fig. 2E-J). In situ hybridization for EBV (EBER-ish) was negative in follicular B-cells and predominantly positive in the larger CD4 positive T-cells in the paracortex (Fig. 2, K). Based on the histologic, immunohistochemical and EBER-ish findings along with the clinical history of one year of persistent IM symptoms following an acute EBV infection in an immunocompetent individual meeting the diagnostic criteria for HLH without genetic predisposition, the essential and two of the desirable diagnostic criteria for SEBVTCL evolved from CAEBV as described in WHO-HAEM5bv were met. More specifically, the essential diagnostic criteria of tissue infiltration by atypical, EBV infected, CD4 positive T-cells in an immunocompetent patient with fever are met, and the desirable criteria of HLH and hepatosplenomegaly are met [3, 5].

**Fig. 3:**
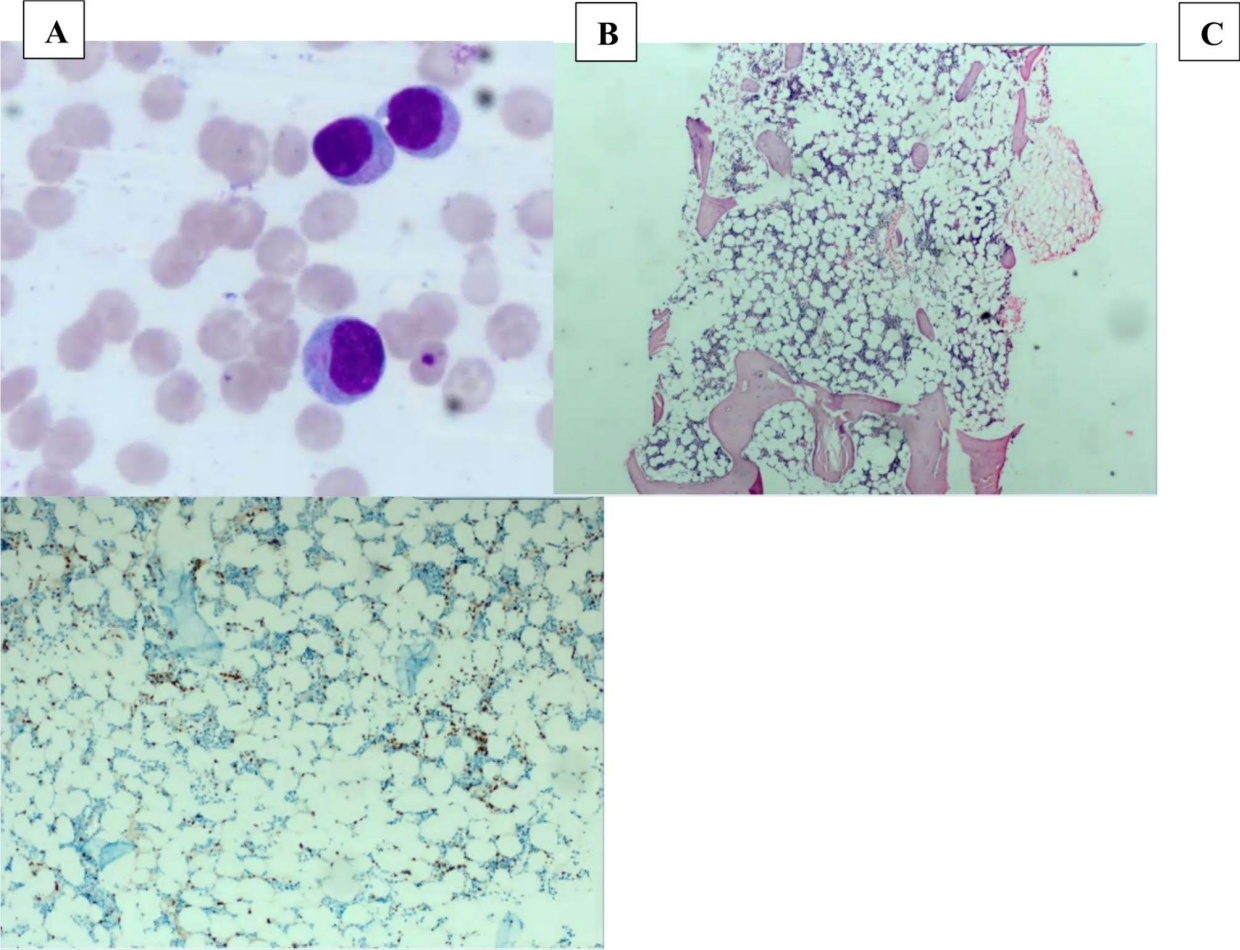
Wright Giemsa stained blood smear, H&E stained marrow core, CD3 stain marrow core. Predominance of intermediate sized lymphocytes with granular cytoplasm in blood (**A**); hypocellular bone marrow without focal lymphoid aggregates (**B**); scattered CD3 positive small T-cells in bone marrow (**C**)

Review of a peripheral blood smear showed a predominant population of monomorphous atypical lymphoid cells with condensed nuclear chromatin, and basophilic cytoplasm with fine azurophilic granules (Fig. 3). Bone marrow core sections showed markedly hypocellular bone marrow for age (10% cellular) with trilineage hematopoietic maturation, and no lymphoid aggregates confirmed by CD3 immunostaining which showed few, scattered small T-lymphocytes (Fig. 3).

**Fig. 4:**
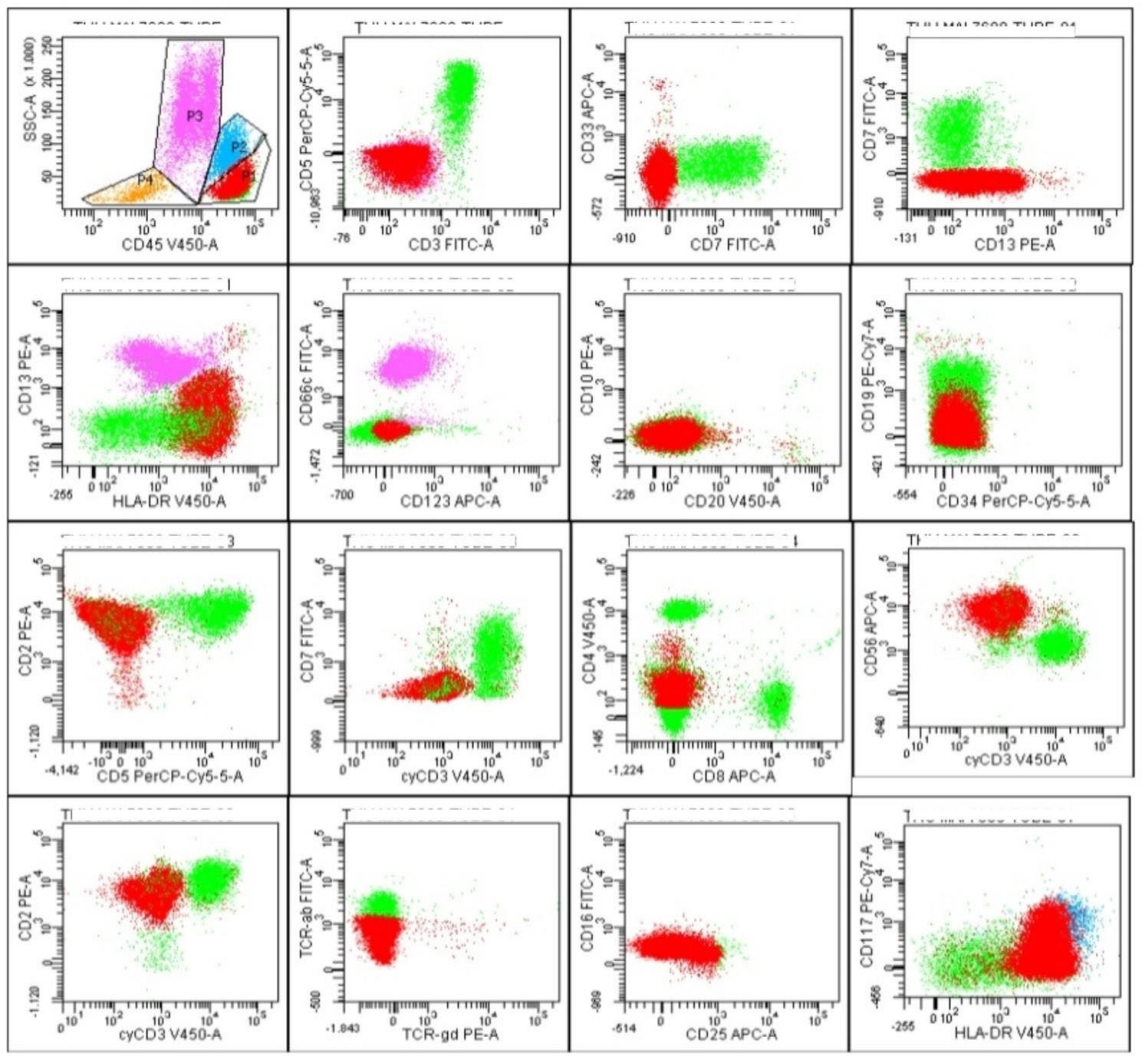
Flow cytometric immunophenotyping gated with FACS Diva software showing predominant atypical NK cell population expressing CD2 and CD56 but lacking surface CD3 and T-cell receptors

Flow cytometric immunophenotyping performed on blood (total leukocyte count 7.52 k/ul) revealed that more than 60% of leukocytes were atypical NK cells expressing surface CD2 and CD56 but lacking surface or cytoplasmic CD3, other T-cell antigens, or T-cell surface receptors (Fig. 4). The blood smear morphology and the flow cytometric immunophenotyping study together meet the WHO-HAEM5 essential diagnostic criteria for ANKL. Specifically, there is a systemic proliferation of atypical NK cells lacking surface T-cell receptors (TCR) in a patient who presents acutely with fever and systemic symptoms [2].

## Laboratory and clinical follow-up

When preliminary laboratory testing confirmed the clinical suspicion for HLH, and after the lymph node, bone marrow and blood specimens were obtained, a trial of intravenous dexamethasone was initiated with rapid resolution of fever, but with minimal to no change in blood counts, adenopathy or organomegaly. When the hematopathology diagnoses of synchronous SEBVTCL and ANKL with literature review, including diagnostic criteria from WHO-HAEM5bv, were presented to the clinical physicians, with assistance from a board certified academic hematopathologist from the United States (VJD), a decision was made to treat the patient with the SMILE (dexamethasone, methotrexate, ifosphamide, L-asparaginase, etoposide) protocol [7]. After the second course of SMILE, the lymphadenopathy and hepatosplenomegaly were improved on CT scan. Blood morphology and flow cytometric immunophenotyping examination revealed decreased fraction and total numbers of circulating atypical NK cells, comprising 2% of circulating lymphocytes and less than 1% of total leukocytes. After the third cycle of SMILE, blood EBV measured by PCR was reduced to 13,650 copies/mL. The plan was to complete four courses of SMILE, restage, and perform allogeneic peripheral blood stem cell transplantation when clinically appropriate, however, during the fourth cycle the patient developed bacterial pneumonia requiring ventilator support necessitating cessation of chemotherapy. Despite good response to antibiotics, the patient refused all further treatment, requesting that she be allowed to return home.

## Discussion and review of the literature

To the best of our knowledge, this is the first reported case in the English language literature of what may be a synchronous presentation of SEBVTCL and ANKL in an adult patient presenting with acute HLH and prior clinical history consistent with CAEBV. Most importantly, all the laboratory studies described here were performed in a hospital laboratory in Vietnam. The images in Figs. 1 and 2 are certainly not of the highest quality. They are screenshots taken from a personal laptop computer during a real-time video conference with a Vietnam hospital pathologist, where sub-optimally fixed, sub-optimally stained biopsy preparations were reviewed and compared to the clear digital images illustrated in WHO-HAEM5bv. Diagnostic modalities beyond what is required for these diagnoses in WHO-HAEM5bv, such as dual staining for CD4 and EBER, LMP, and EBNA2 in the lymph node are not currently available in Vietnam.

Facility of hematopathology diagnosis is limited in Vietnam due to lack of specific training in hematopathology for Vietnam pathologists, lack of readily available expert consultation services, and lack of consistent quality control for diagnostic specimen preparations [8]. Diagnoses for this patient were expedited due to a multi-year commitment made by the admitting institution to work with a US-based nonprofit and its volunteers in histopathology, immunohistochemistry, and flow cytometry methods. Diagnostic slides along with clinical data and other studies were reviewed with a US-based academic hematopathologist using a secure, web-based video conferencing software, Zoom™, made available to the faculty of the University of Minnesota Medical School. Although use of video conferencing software for sharing pathology cases became quite common in the US during the COVID19 pandemic restrictions, it is not new or unproven [9].

Diagnosing SEBVTCL and distinguishing it from CAEBV has been simplified in WHO-HAEM5bv. Diagnostic recognition of both entities has been impeded in the past not just by their perceived scarcity but by a rapid evolution in our understanding of EBV driven lymphoid proliferations followed by continued updating of nomenclature for their classification. A large series postulating the existence of a chronic infection with EBV was published in the United States in the 1980’s. That study included 23 adult patients with IM symptoms that persisted for up to 22 years after acute infection with EBV as identified by serologic testing [10]. Over the next two decades, several reports were published by Japanese researchers describing children with a persistent increase in EBV-positive/CD4-positive T-lymphocytes detected in their blood either by dual immunofluorescence or a combination of techniques including CD4 lymphocytes concentrated using flow cytometry with cell sorting and EBV DNA detection by in-situ hybridization. The Japanese reports described these children with chronic IM symptoms and EBV infected CD4 lymphocytes as progressing to a deadly EBV-positive T-cell lymphoma [11–13]. Notably, as recently as 2011, a 28-year, multi-institutional retrospective review of CAEBV cases diagnosed in the United States included only 19 total patients, with 11 of those manifesting an EBV positive B-cell proliferation [14]. Not long after that retrospective study was published, a revised 4th edition of the WHO classification defined “CAEBV” as being solely a T/NK-cell lymphoproliferative disorder with revised diagnostic criteria that included both clinical and pathologic findings [15].

All entities listed in the 5th edition of the WHO Classification provide “Essential” and “Desirable” criteria for each pathologic diagnosis [1]. The essential diagnostic criteria for CAEBV now include the clinical finding of IM symptoms persisting for greater than three months, the finding of increased EBV DNA in peripheral blood or tissue demonstration of EBV RNA, and exclusion of known immunodeficiency, autoimmune disease, or malignancy. The single essential criterion distinguishing SEBTCL from CAEBV in WHO-HAEM5bv is the presence of atypia in the EBV infected T-cells; clonal T-cell receptor gene rearrangements are acknowledged as being present in both CAEBV and SEBTCL. WHO-HAEM5bv has categorized both CAEBV and SEBVTCL under the heading of, “EBV-positive T-cell and NK-cell lymphoid proliferations and lymphomas of childhood.” As our case suggests, supported by other reports, these entities can and should be diagnosed, when appropriate, in adults [16, 17].

The definition for Aggressive NK-cell leukemia is unchanged in WHO-HAEM5bv, however, the new essential diagnostic criteria that are provided simplify making the diagnosis. ANKL is generally thought to be rare, more common in Asians, and to have very poor prognosis with death occurring within months of diagnosis [2]. Two recently published retrospective data reviews suggest that allogeneic hematopoietic stem cell transplant may provide durable remissions in some patients, while also shedding some light on the incidence and epidemiology of the disease. A review of data from the Japan Society for Hematopoietic Cell Transplantation identified 59 pediatric and adult patients transplanted for a diagnosis of ANKL over 19 years. A 14-year retrospective review of data from the Center for International Blood and Marrow Transplant Research, a registry that includes only adults treated at one of more than 500 transplant centers worldwide, identified 21 patients with ANKL diagnosis confirmed by a hematopathologist. 15 of those 21 patients (71%) were classified as Caucasians [18, 19]. As these are retrospective reviews, it is not possible to exclude biases due to the historical experience of Japanese pathologists with the diagnosis of these entities, and the possibility that Western pathologists are less experienced at recognizing them.

Both SEBVTCL and ANKL are suspected to evolve from CAEBV, as they appear to be in the patient reported herein. This would seem to heighten the need for accurate identification of CAEBV so that evolution to more aggressive disease can be diagnosed promptly and managed with optimal therapy. This would require both clinicians and pathologists to be aware of CAEBV and its diagnostic criteria. Clinicians seeing patients with persistent mononucleosis-like symptoms would need to order EBV serologies and, if consistent with prior infection, then order blood PCR for EBV. Pathologists would need to include CAEBV in their differential diagnosis for lymph nodes showing paracortical hyperplasia with small to intermediate-sized T-lymphocytes, then stain for EBV RNA and, if identified, confirm that there is no clinical history of immunodeficiency, autoimmune disease, or malignancy.

In conclusion, we present an adult Vietnamese patient who was rapidly diagnosed with, and promptly and appropriately treated for, synchronous HLH, SEBVTCL, and ANKL all of which likely evolved from CAEBV that was suspected clinically and closely followed by primary care physicians in a country with limited, but improving, access to diagnostic resources. This patient outlived the expected prognosis for any one of these diseases due to rapid recognition and diagnosis made possible by Vietnam’s emerging culture of collaboration between clinicians and pathologists, laboratory quality improvement, and continuing pathology education, as well as by assistance from laboratory volunteers from a US academic health center. Most remarkably, all laboratory testing was performed in Vietnam with real-time assistance using video conferencing software. Diagnoses made using the provisional WHO 5th edition classification must be recognized and embraced by the global community, as hesitancy in accepting these diagnoses could inadvertently marginalize the innovative ideas and perspectives from emerging economies. Embracing these contributions is integral to realizing the WHO goal of creating an inclusive, dynamic, and compassionate global health community. Moreover, ignoring insights drawn from the beta version of the WHO 5th edition could lead to a lost opportunity in enhancing medical understanding across diverse socio-economic landscapes.

## Data Availability

for telepathology and laboratory resources, including molecular genetic testing, provided by Ho Chi Minh City, Vietnam, Blood Transfusion and Hematology Hospital Office of Clinical Research (https://bthh.org.vn).

## Abbreviations

EBV: Epstein Barr Virus
ANKL: Aggressive NK cell leukemia
SEBVTCL: Systemic EBV positive T,cell lymphoma
HLH: Hemophagocytic lymphohistiocytosis
PCR: Polymerase Chain Reaction
CAEBV: Systemic chronic active EBV disease
WHO,HAEM5bv: World Health Organization Classification of Haematopoietic Neoplasms 5th edition beta version online
SMILE: Chemotherapy protocol including dexamethasone, methotrexate, ifosphamide, L,aspariginase, etoposide
